# Initiating buprenorphine to treat opioid use disorder without prerequisite withdrawal: an updated systematic review

**DOI:** 10.1186/s13722-025-00548-z

**Published:** 2025-02-20

**Authors:** Kathleen K. Adams, Kristin Waters, Diana M. Sobieraj

**Affiliations:** University of Connecticut School of Pharmacy, 69 N. Eagleville Rd. Unit 3092, Storrs, CT 06269 USA

**Keywords:** Buprenorphine, Low dose buprenorphine induction, Opioid use disorder, Microdosing, Opioid withdrawal, Bernese method

## Abstract

**Background:**

Withdrawal prior to buprenorphine initiation may be intolerable or create barriers to therapy. We aim to update our previous systematic review on the efficacy and safety of buprenorphine initiation strategies that aim to omit prerequisite opioid withdrawal (POW).

**Methods:**

We used the same search strategy for this update as in the original review with the modification of an additional term “low dose.” We searched Embase and Scopus from April 11, 2020 to August 1, 2024 with searches in Google Scholar and www.clinicaltrials.gov. A study was included if it described patients with opioid use disorder or chronic pain that transitioned from a full mu-opioid agonist to buprenorphine without preceding withdrawal and reported withdrawal during initiation as an outcome. Two investigators independently screened citations and articles for inclusion, collected data using a standardized data collection tool, and assessed study risk of bias.

**Results:**

Forty-four articles met our inclusion criteria; 31 were case reports/series reporting 84 cases and 13 were single-arm observational studies reporting a total of 576 cases. These studies were added to the literature from our original systematic review, totaling 59 studies and 682 patients. Sublingual buprenorphine was the most common initial formulation, comprising 55% (376/682) of cases. In case reports/series, use of a validated scale to measure withdrawal was uncommon; validated scales were only used in 36% of patients. All other patients had withdrawal assessed in a manner not utilizing a validated scale. Approximately half of these patients experienced any level of withdrawal (57/106 = 54%). The specific outcome of “any level of withdrawal” was not consistently reported in single-arm observational studies. Eight studies reported on any level of withdrawal, which occurred in 41% (177/428) of initiation attempts; some patients experienced more than one initiation attempt. Thirteen patients in case reports/series and 37 patients in the single-arm observational studies reported clinically significant withdrawal (50/682 = 7%). 81% (451/555) of patients transitioned to buprenorphine.

**Conclusion:**

The prevalence of buprenorphine dosing strategies that aim to omit POW has vastly increased over the past 4 years. While quality of evidence remains low, the increased quantity of publications and integration into health-system guidelines and protocols demonstrates the need for prospective, controlled studies. It is unknown how selection bias impacts current findings, further highlighting the need for prospective, randomized, controlled trials evaluating these dosing strategies.

**Supplementary information:**

The online version contains supplementary material available at 10.1186/s13722-025-00548-z.

## Introduction

Buprenorphine is a first-line treatment for opioid use disorder (OUD) that reduces mortality and use of illicit opioids [1, 2]. Historically, a major barrier for buprenorphine treatment has been the prerequisite opioid withdrawal (POW) prior to initiation [3]. Since the publication of the “Bernese method” by Hämmig et al. in 2016, clinicians have developed and modified various frameworks of alternative buprenorphine dosing strategies [4]. “Buprenorphine microdosing,” also termed “low dose buprenorphine initiation (LDBI)” describes nontraditional buprenorphine dosing strategies that aim to omit POW during buprenorphine initiation [3]. In using these alternative strategies, some patients can continue their full opioid agonists without experiencing withdrawal [5]. While registered clinical trials (NCT04893525, NCT05450718, NCT04234191, NCT05644587, NCT05307458, NCT05944952) may inform future practice, there are currently no prospective randomized controlled trials comparing LDBI to traditional buprenorphine initiation. Despite limited evidence and lack of consensus guidelines, healthcare institutions are frequently using these strategies and developing protocols [6]. Patients with OUD are increasingly aware of and interested in obtaining access to treatment with buprenorphine without undergoing highly uncomfortable opioid withdrawal [7]. Buprenorphine treatment, including LDBI, has become more complicated by the presence of high-potency synthetic opioids (HPSO) such as fentanyl in the illicit drug supply. Higher doses of buprenorphine may be required for patients exposed to HPSO to achieve stabilization [8]. We aim to provide an update to our previous systematic review evaluating alternative buprenorphine initiation strategies that aim to omit POW [9]. 

## Methods

### Data sources and search

We used the same search strategy for this update as in the original review with the modification of an additional term “low dose.” [9] Since initial publication, the term “low dose buprenorphine initiation” has emerged and is preferred to “microdosing,” [10] We searched Embase and Scopus from April 11, 2020 to August 1, 2024. To augment our bibliographic database search, we searched Google Scholar on January 31, 2024 and reviewed potentially relevant citations from the prior year as well as forward citation tracking of relevant citations. We reviewed the references of included studies. Finally, we searched www.clinicaltrials.gov for completed studies with results or ongoing studies to evaluate results that may inform future practice.

### Study selection

Two investigators independently screened the title and abstract of each citation identified by the search and subsequently reviewed the full-text manuscript for final inclusion. Discrepancies were resolved by discussion or a third investigator. The inclusion criteria for this update were consistent with the original systematic review, with the exception that we excluded studies transitioning from kratom and tapentadol as literature has suggested these agents act as atypical opioids as opposed to true full mu opioid agonist [11, 12]. Studies of any design were included if they (1) evaluated an alternative buprenorphine initiation strategy that aimed to avoid POW, (2) was in patients with substance use disorder and/or chronic pain that were taking a full mu opioid agonist and (3) reported the presence or absence of withdrawal during the initiation phase. Abstract-only publications were not included.

We defined alternative strategies that aimed to avoid POW as those that either (1) overlapped the full opioid agonist and buprenorphine (omitting the opioid free period and thus omitting POW) or (2) reported a baseline Clinical Opiate Withdrawal Scale (COWS) score of less than 5 (demonstrating lack of withdrawal symptoms). In the absence of a reported baseline COWS score for case reports only, we estimated the maximal possible score using symptoms reported prior to the first buprenorphine dose and included case reports with a score less than 5. If papers described overlap of full opioid agonists with buprenorphine but there were indicators that the patient was in withdrawal, these were excluded. In addition to withdrawal, additional outcomes of interest included severity of withdrawal, number of patients that transitioned from full opioid agonist to buprenorphine, and duration of the initiation period.

### Data extraction and risk of bias assessment

Using a standardized data collection tool, two investigators independently collected the following data from included studies: patient characteristics including age, sex, substance use history, indication for buprenorphine; initiation regimen characteristics including the method used, setting, medication formulation and dosing details, duration of initiation period; and information to assess risk of bias and our outcomes of interest.

We categorized publications describing fewer than 10 cases as case reports/case series while publications describing 10 or more cases were categorized as single-arm observational studies [13]. Dosing strategies are described based on the initial dosage form used in the protocol.

We assessed the internal validity of included case reports/case series using the tool by Murad et al., consistent with the original systematic review [14]. The tool includes eight questions across four domains: selection, ascertainment, causality and reporting. We answered each question as “yes” or “no” and summarize assessments. We omitted two of the original eight questions regarding challenge/re-challenge phenomenon and dose–response relationships intended for reporting of adverse events because this was not applicable to our topic. Internal validity was assessed for each study by two separate investigators with conflicts resolved through discussion.

To assess the risk of bias of single-arm observational studies, we used the MINORS tool [15]. This tool addresses 12 unique components of internal validity for non-randomized comparative studies. Each study was evaluated by two independent reviewers and each of the 12 components was scored by the reviewer as 0 (not reported), 1 (reported but inadequate), or 2 (reported and adequate). The two reviewers then reconciled discrepancies to arrive at a final judgement. The protocol for this systematic review was not published.

## Results

Upon searching, we identified 1308 citations after duplicates were removed (Additional File 1 _ Search Strategy). After citation screening, we reviewed 89 articles at the full text level. Forty-four articles met our inclusion criteria; 31 were case reports/case series reporting 84 cases and 13 were single-arm observational studies reporting 576 cases totaling 660 new cases in the updated search. These studies were added to 22 cases across 15 publications from our original systematic review, totaling 59 studies (Additional File 2 _ Study Selection) and 682 patients; two patients from our original systematic review were removed due to the updated exclusion criteria of kratom and tapentadol.

### Quality assessment

We evaluated risk of bias for each of the 31 new case reports/case series (see Additional File 3, Appendix Table 1). The domains of selection, exposure ascertainment, outcome ascertainment, and causality had the most weaknesses. Four (13%) of the articles described selection methods. All reports included an adequate length of follow-up. No reports adequately ruled out potential alternative causes of withdrawal because they either provided supportive medications that could have influenced withdrawal symptoms or did not comment whether supportive medications were given or not. Sixteen (52%) articles used methods, such as medical records or validated tools, to ascertain the outcome of withdrawal. Eighteen (58%) utilized medical records or direct observation as opposed to patient reported information to confirm that the patient took medications as prescribed. Twenty-five (81%) articles reported cases with sufficient detail for replication.

Additionally, we evaluated risk of bias for each of the 13 single-arm observational studies (see Additional File 3, Appendix Table 2). The domains of unbiased assessment of study endpoint and prospective calculation of study size had the most weaknesses; no studies completed a prospective study size calculation. Most studies reported on a follow up period that was appropriate to sufficiently capture withdrawal outcomes (85%) as well as executed prospective collection of data (85%). Most studies (77%) reported no significant loss to follow-up. Use of validated tools, such as COWS, to measure our primary outcome of withdrawal were inconsistent and only 38% of papers provided an unambiguous explanation of criteria used to evaluate withdrawal. Clearly stated aim and inclusion of consecutive patients occurred for most, but not all studies.

### Patient characteristics

Nearly all cases came from the United States or Canada and represented both sexes ages 16–73 years old (See Additional File 3; Tables 1 and 2). Most patients had OUD. Any history of heroin or fentanyl was described in 58% (62/106) of patients in case reports/case series. When reported, fentanyl or heroin use was identified in the majority of patients in single-arm observational studies; not all articles reported this (See Additional File 3, Table 3). 42% (45/106) of patients in case reports/case series were taking methadone when they underwent buprenorphine initiation while 23% (24/106) of patients were taking illicit fentanyl/heroin. Opioid regimens immediately prior to buprenorphine initiation were varied among single-arm observational studies. These findings align our original review in that fentanyl/heroin continues to be common and these alternative dosing strategies are being used for a wide variety of patients taking various types of full opioid agonists.

### Dosing strategy characteristics

The most commonly used initial buprenorphine formulation in the published literature was sublingual, comprising 55% (376/682) of cases. Other routes of buprenorphine administration included 13% transdermal (88/682), 14% intravenous (95/682), and 18% buccal (123/682). This contrasts with our original review as our previous publication did not include any strategies that utilized intravenous or buccal formulations.

Among case reports/case series, median time to completion of initiation was 9 (6–11) days with buccal, 6 (5–25) days with intravenous, 10 (4–16) days with transdermal patch, and 8 (3–120) days with sublingual. Concomitant full opioid agonists were administered for the majority of time it took to initiate and increase the buprenorphine dose. This overlap was approximately 7 days for most described dosing strategies.

Methods to report time to completion varied between case reports and single-arm observational studies (See Additional File 3; Table 4). In lieu of exact timelines, single-arm observational studies typically reported a protocol that patients followed with opportunities for adjustments. Some studies used lower buprenorphine dose cut offs such as 4 or 8 mg as their terminal dose for evaluation. It was uncommon for studies to report patients achieving a dose of at least 24 mg. While protocols varied in length, they generally ranged between 3 and 8 days. However, some patients required much longer to transition to buprenorphine. Most protocols reported overlap with full opioid agonists for the majority of the time that buprenorphine was titrated. These findings align our original review that found that median time to completion was approximately 1 week.

### Withdrawal

Our primary safety outcome of interest was the number of cases that experienced withdrawal during initiation. Current sources of evidence varied in how withdrawal was reported. In case reports / case series, use of a validated scale to measure withdrawal, such as COWS or Subjective Opiate Withdrawal Scale (SOWS), was uncommon and only occurred in 36% of patients. All other patients had withdrawal assessed in a manner not utilizing a validated scale. Approximately half of these patients experienced any level of withdrawal (57/106 = 54%).

The specific outcome of “any level of withdrawal” was not consistently reported in single-arm observational studies. “Any level of withdrawal” aimed to capture participants that may have experienced any severity of withdrawal (minimal, mild, moderate, severe) during initiation. Studies were considered to report on “any level of withdrawal” if they provided some degree of patient-level data on the range of withdrawal severities experienced in their study. While some level of withdrawal reporting had to occur to be included in our study, methods for reporting varied between studies. For example, some studies only reported if patients experienced precipitated withdrawal while other studies reported withdrawal outcomes in aggregate, precluding the ability to calculate rates of minimal or mild withdrawal across all studies. Not all studies included data on the spectrum of withdrawal severities that can be seen during initiation, which we aimed to capture with “any level of withdrawal.” Eight studies reported any level of withdrawal, which occurred in 41% (177/428) of initiation attempts; some patients experienced more than one initiation attempt. Reporting methods included patient reported symptoms, progress note review, and COWS. This finding aligns with our previous review which found 14 of the 24 cases (58.3%) reported any withdrawal symptoms.

Three studies reported aggregate COWS scores. Adams et al. reported median maximum COWS of 7 (1–18) in the 24/45 patients that had documented COWS scores. Hayes et al. reported median maximum COWS for successful initiations as 1 (IQR 0–2) and median maximum COWS for unsuccessful initiations as 5 (IQR 1–12). Murray et al. reported mean COWS scores as 1.6 (SD 2.6) on day 5 for all patients; overall mean COWS scores did not exceed 3.5. While any withdrawal seems common in these reports, it appears to be mild in severity.

Two studies did not report COWS and instead noted precipitated withdrawal, which occurred in 2% (2/95) of patients. However, 1 additional patient in this cohort discontinued due to persistent withdrawal.

We considered withdrawal to be clinically significant if it was rated as moderate or severe, precipitated withdrawal, or led to treatment cessation. Using this definition allowed us to combine findings across study types. Thirteen patients in case reports/case series and 37 patients in the single-arm observational studies reported clinically significant withdrawal (50/682 = 7%); a similarly low rate was found in the original systematic review.

### Transition to buprenorphine

76% (80/105) of patients in case reports/case series transitioned to buprenorphine monotherapy. An additional 13 patients continued use of full opioids agonists with concurrent buprenorphine meaning that 89% (93/105) of all patients transitioned to buprenorphine with or without concurrent agonists. One patient was still undergoing buprenorphine and full opioid agonist cross-taper at the time of manuscript submission and did not state if the patient transitioned. Twelve (11%) patients did not transition to buprenorphine. Of the 12 patients that did not transition to buprenorphine, five experienced at least moderate withdrawal and/or withdrawal leading to treatment cessation. Other reasons included side effects such as oversedation and nausea, buprenorphine feeling ineffective for pain, or loss to follow up.

In single-arm observational studies, 80% (358/450) of patients transitioned to buprenorphine; one study did not report rates of transition. Studies by Murray, Raheemullah, Bhatraju, and Noel indicated the number of patients that continued full opioid agonists (*n* = 11, *n* = 3, *n* = 23, and *n* = 10 respectively), but it is not clear if participants in the other studies did as well. In the single-arm observational studies, lower rates of transition to buprenorphine were seen in the outpatient setting compared to those seen in case reports; only 62% (44/71) of outpatients transitioned to buprenorphine in single-arm observational studies. In contrast, 82% of outpatients (55/67) transitioned to buprenorphine in case reports.

In this updated review, 57 of the 59 included studies reported transition rates to buprenorphine. Of those 57 studies, 81% (451/555) of patients transitioned to buprenorphine with or without full opioid agonists. At least 11% of those patients (60/555) continued concurrent full opioid agonists. This is a reduction in patients transitioning to buprenorphine monotherapy since our original review, where transition to buprenorphine with complete cessation of opioid agonists was achieved in 87.5% (*n* = 21) of cases.

## Discussion

### Summary of findings

This systematic review aimed to evaluate the efficacy and safety of buprenorphine initiation strategies that aim to omit POW. For these two outcomes, we focused on rates and severity of withdrawal as our safety measure and rates of transition to full opioid agonist as our main measure of efficacy. Consistent with our original review, evidence remains limited to uncontrolled observational data. No prospective randomized controlled trials were identified. Given the lack of controlled studies, our interpretation of this data is still limited.

While minimal or mild withdrawal remains common using LDBI strategies, clinically significant withdrawal appears uncommon based on the published literature, occurring in approximately 7% of published cases. This is in stark contrast to traditional dosing that recommends patients experience at least moderate withdrawal (COWS > 12) with consideration of a higher COWS in patient using fentanyl [16]. Replacement of heroin with fentanyl in illicit drug markets may create barriers to buprenorphine initiation [17] and not all patients can tolerate the physical distress required for traditional initiation [4, 18]. This finding suggests that LDBI strategies allow some patients to transition to buprenorphine with less withdrawal than with traditional dosing strategies and that some patients do not experience any withdrawal. These dosing strategies are an additional tool for clinicians in overcoming withdrawal-related barriers for their patients.

A notable difference in this updated review compared to our original review is a lower transition rate to buprenorphine and buprenorphine monotherapy. Our original review captured literature that had been published from 1996 to April 2020. Since that time, HPSO have come to dominate the illicit opioid supply which may have contributed to this lower buprenorphine transition rate [19]. In one retrospective survey of patients entering treatment for OUD, 1163 patients (69.3%) reported either “probably” or “definitely” using fentanyl before entering treatment [20]. Of the 339 patients who reported taking buprenorphine within 24 h of fentanyl, 36.5% reported experiencing severe withdrawal compared to only 15% of those who reported taking methadone within 24 h of fentanyl. Fewer patients reported that buprenorphine completely alleviated opioid withdrawal (38.4%) compared to those treated with methadone (44.3%). In this study, people who used buprenorphine within 24 h of fentanyl had the highest odds of developing severe withdrawal (OR = 5.202, 95% CI = 1.979–13.675, *p* = 0.001), but the odds were still high for those who had received buprenorphine within 48 h of fentanyl (OR = 3.352, 95% CI = 1.237–9.089, *p* = 0.017). While this retrospective review did not capture the method of buprenorphine induction trialed for the patients who experienced severe withdrawal, it highlights the increased likelihood of experiencing withdrawal when buprenorphine is initiated within 48 h of HPSO use. Anecdotal reports from people who use fentanyl also support the need for increased awareness and investigation of how to best transition someone from HPSO to buprenorphine [21]. Although opioid withdrawal is considered non-fatal by clinicians, the agony and pain of withdrawal may serve as a major deterrent to seeking care or to successfully completing a transition to buprenorphine.

However, an 81% transition rate is clinically meaningful in the management of patients with OUD. According to a study published in 2023, less than 25% of patients with OUD receive pharmacologic treatment for the disorder [22]. As the model for treating OUD has increasingly included harm reduction strategies, any engagement in treatment for OUD can have a positive impact on lives and wellbeing of patients [23]. Harm reduction is an approach to the treatment of substance use disorders that aims to reduce adverse consequences associated with substance use for both people who use drugs (PWUD) and for society. This approach includes prevention, risk reduction, and health promotion and does not assume that all PWUD have the same goal, including abstinence [23]. Within the framework of harm reduction, the 81% buprenorphine transition rate is especially clinically meaningful.

In assessing the substantial number of patients that continued full opioid agonists at the end of the reporting period, it is unclear whether this is an intentional co-occurring medication use or if study authors chose to publish what they felt to be the most informative part of the initiation process. For example, some articles did use 4 mg or 8 mg as their terminal dose for evaluation. It is important to note that while the dose of 16 mg had previously been associated with long term retention [24] recent data has suggested higher doses of 24 mg may be necessary for improving retention in the era of fentanyl [25]. While not all studies reported terminal buprenorphine doses, the majority reported a final dose less than 24 mg. This suggests additional buprenorphine titration outside of the study period could potentially be occurring. As a result, time to complete these strategies could be longer than the approximate 1 week that is suggested in the literature. While there are some exceptions, these dosing protocols generally take longer than traditional buprenorphine initiation, which is usually completed in 1–2 days [16]. It is unknown if there is a positive, negative, or neutral impact in extending the time it takes for initiation and may vary based on patient factors.

We noted a discrepancy between inpatient and outpatient transition rates in single-arm observational studies. This may be due to the higher potential for loss to follow up in the outpatient setting and perhaps selection of successful cases when submitting case reports for publication. In the hospital, clinicians have the benefit of reliable medication administration and ease of continuous monitoring. Higher doses of opioids may also have been used to manage pain conditions in the hospital.

While intravenous and buccal formulations have been introduced to the literature since the original review, the sublingual dosage form remains the most commonly reported strategy in the literature. This may be because the sublingual form was used in the first published dosing strategies and can be used in both the inpatient and outpatient settings. However, this finding is in contrast to a recent survey by Hardy and colleagues, which suggests that buccal may be more commonly used in hospital practice [6]. 

Lastly, we note the exponential increase in publications over the past few years. Our update includes nearly thirty times as many cases as our first systematic review. This illustrates that, despite the lack of prospective data to inform practices, clinicians continue to use and refine these strategies and practice with them will likely not abate. As such, it is critical that there is support for prospective trials in this area of research.

### Limitations

The rate of successful buprenorphine initiation may be overstated in the literature due to publication bias. Positive results for novel ideas are intriguing, citable, and papers are more likely to be published if the reported results are positive [22]. As novel ideas become intertwined into traditional clinical practice, opportunity emerges to contribute unsuccessful outcomes to the literature, allowing clinicians to share best practices. We aimed to include all study types, however the literature base still lacks prospective randomized controlled trials. Our synthesis was limited to case reports/case series and single-arm observational studies, which were inherently suffer from selection bias. Some studies reported choosing only the successful cases in their publications. Many studies did not differentiate between people who had used HPSO prior to initiation attempt, which may impact success rates. Our primary safety outcome, withdrawal, varied in how it was assessed and measured across publications. When validated tools, such as COWS or SOWS, were not used, the authors were limited to patient and provider reports, thus limiting our analysis. While a number of additional publications do exist evaluating LDBI dosing strategies, those that did not report the presence or absence of our primary outcome, withdrawal, were not included. This may have impacted our findings with regards to transition rate; this rate may have been different if transition rate was used as our primary outcome instead of withdrawal. Additionally, due to the limited number of studies and varying methodologies and reporting structures, we are unable to draw conclusions in comparing strategies based on dosage form or protocol characteristics. Finally, long-term retention is not frequently reported and thus potential long-term outcomes of omitting POW with LDBI strategies are unknown. While our systematic review provides substantial insight into the reported withdrawal and transition outcomes seen in LDBI strategies, due to the lack of prospective randomized controlled trials and low-quality evidence, we are still unable to make direct comparisons to traditional initiation strategies. While LDBI strategies appear successful, the literature base precludes the ability to suggest LDBI over traditional dosing or make specific conclusions about what patient should receive which dosing strategy. Because of the literature base, it is also not possible to delineate treatment outcomes based on specific patient factors. Configurational analysis has previously been used to predict withdrawal outcomes based on patient factors and may be useful in future studies [26]. 

Our study differs from previous narrative and systematic reviews in that we only included studies that reported withdrawal as an outcome; we did not include studies that describe LDBI without reporting withdrawal. We chose this methodology since the purpose of LDBI strategies is to omit the need for prerequisite withdrawal and withdrawal is known barrier to buprenorphine initiation [27–30]. 

## Conclusion

The prevalence of buprenorphine dosing strategies that aim to omit POW in the literature has vastly increased over the past 4 years. While quality of evidence remains low, the quantity of publications in the literature and integration into health-system guidelines and protocols begs the need for prospective, controlled studies. While minimal or mild withdrawal symptoms are commonly reported, few published studies report clinically significant withdrawal and most patients transition to buprenorphine. These strategies are expected to take longer than traditional buprenorphine initiation. It is unknown how selection bias impacts current findings, and prospective, randomized, controlled trials evaluating these dosing strategies compared to traditional initiation are urgently needed.

## Electronic supplementary material

Below is the link to the electronic supplementary material.

Additional file 1: Search strategy (Search strategy for systematic review)

Additional file 2: Study selection (Database screening flowsheet)

Additional file 3: Tables (**Table 1**: Patient characteristics in case reports/case series. **Table 2**: Summary of dosing strategy characteristics from case reports/case series. **Table 3**: Summary of patient characteristics from single-arm observational studies. **Table 4**: Summary of dosing strategies from single-arm observational studies **Appendix Table 1**: Risk of bias summary of case reports/case series. **Appendix Table 2**: Risk of bias summary of single-arm observational studies. **Appendix Table 3**: Additional case report/observational study references. **Appendix Table 4**: Case reports/case series summary table. **Appendix Table 5**: Single arm observational studies summary table.)

## Data Availability

Datasets used and/or analyzed during the systematic review are available from the corresponding author on reasonable request.

## Abbreviations

COWS: Clinical Opiate Withdrawal Scale
OUD: Opioid Use Disorder
POW: Precipitated Opioid Withdrawal
SOWS: Subjective Opiate Withdrawal Scale
