# Supplementary material for: Initiating buprenorphine to treat opioid use disorder without prerequisite withdrawal: an updated systematic review
Source: Addict Sci Clin Pract. 2025 Feb 20;20:19. doi: 10.1186/s13722-025-00548-z (PMC11841166; doi:10.1186/s13722-025-00548-z)
Supplement: Supplementary file 1 — Additional file 1: Search strategy (Search strategy for systematic review) [file 13722_2025_548_MOESM1_ESM.doc]

**Scopus – 317 results – 8/1/2024**

(TITLE-ABS(buprenorphine OR acimaphin OR acinorphin OR addictex OR addnok OR algesalona OR anorfin OR astec OR belbuca OR brixadi OR bugnanto OR bunondol OR bunorfin OR bunov OR bupacal OR bupainx OR bupalster OR bupan OR bupeaze OR bupensan OR buphin OR buplab OR buplast OR bupramyl OR “bupre-hexal” OR buprefarm OR buprel OR bupremyl OR buprenaddict OR buprenal OR buprenex OR buprenocan OR buprenoratiopharm OR buprenorfin OR buprenorfina OR buprenorfine OR buprenorphin OR buprenorphine OR “buprenorphine hydrochloride” OR bupretec OR buprex OR buprine OR busette OR busiete OR butec OR butrans OR buvera OR buvidal OR dolotec OR durlevatec OR feliben OR finibron OR hapoctasin OR laribon OR lepetan OR melodyn OR mitoren OR molterfin OR natzon OR nimedol OR noprex OR norfinox OR norphin OR norspan OR norvipren OR panitaz OR pentorel OR prefibin OR prefin OR prekisan OR prenorvine OR prenotrix OR probuphenine OR probuphine OR ramatrix OR ravata OR reletrans OR relevtec OR sevodyne OR sixmo OR somnena OR sovenor OR sublocade OR subutex OR temgesic OR tephine OR thorbup OR transtec OR tranzileve OR triquisic)

AND

TITLE-ABS(((opiate* OR opioid* OR opium) W/2 (addict* OR depend* OR epidemic* OR crisis)) OR ((opiate* OR opioid* OR opium) W/2 (substitut* OR replace*)) OR (heroin W/2 (addict* OR depend*)) OR “heroinism”)

AND

TITLE-ABS(initiat* OR microdos* OR induc* OR bridg* OR replac* OR substitut* OR (low W/2 dos*))
 AND (PUBYEAR > 2019 AND PUBYEAR < 2025))
 OR
 (AUTHKEY(buprenorphine OR acimaphin OR acinorphin OR addictex OR addnok OR algesalona OR anorfin OR astec OR belbuca OR brixadi OR bugnanto OR bunondol OR bunorfin OR bunov OR bupacal OR bupainx OR bupalster OR bupan OR bupeaze OR bupensan OR buphin OR buplab OR buplast OR bupramyl OR “bupre-hexal” OR buprefarm OR buprel OR bupremyl OR buprenaddict OR buprenal OR buprenex OR buprenocan OR buprenoratiopharm OR buprenorfin OR buprenorfina OR buprenorfine OR buprenorphin OR buprenorphine OR “buprenorphine hydrochloride” OR bupretec OR buprex OR buprine OR busette OR busiete OR butec OR butrans OR buvera OR buvidal OR dolotec OR durlevatec OR feliben OR finibron OR hapoctasin OR laribon OR lepetan OR melodyn OR mitoren OR molterfin OR natzon OR nimedol OR noprex OR norfinox OR norphin OR norspan OR norvipren OR panitaz OR pentorel OR prefibin OR prefin OR prekisan OR prenorvine OR prenotrix OR probuphenine OR probuphine OR ramatrix OR ravata OR reletrans OR relevtec OR sevodyne OR sixmo OR somnena OR sovenor OR sublocade OR subutex OR temgesic OR tephine OR thorbup OR transtec OR tranzileve OR triquisic)

AND

AUTHKEY(((opiate* OR opioid* OR opium) W/2 (addict* OR depend* OR epidemic* OR crisis)) OR ((opiate* OR opioid* OR opium) W/2 (substitut* OR replace*)) OR (heroin W/2 (addict* OR depend*)) OR “heroinism”)

AND

AUTHKEY(initiat* OR microdos* OR induc* OR bridg* OR replac* OR substitut* OR (low W/2 dos*))

AND (PUBYEAR > 2019 AND PUBYEAR < 2025))

**Embase – 1,197 results – 8/1/2024**

('buprenorphine'/exp OR '17 (cyclopropylmethyl) 18 (2 hydroxy 3, 3 dimethylbutan 2 yl) 6 methoxy 18, 19 dihydro 4, 5 epoxy 6, 14 ethenomorphinan 3 ol' OR '17 (cyclopropylmethyl) alpha (1, 1 dimethylethyl) 4, 5 epoxy 18, 19 dihydro 3 hydroxy 6 methoxy alpha methyl 6, 14 ethenomorphinan 7 methanol' OR '17 cyclopropylmethyl 4, 5alpha epoxy 7alpha (1 hydroxy 1, 2, 2 trimethylpropyl) 6 methoxy 6, 14 ethanomorphinan 3 ol' OR '2 (n cyclopropylmethyl 4, 5alpha epoxy 3 hydroxy 6 methoxy 6, 14 ethanomorphinan 6alpha yl) 3, 3 dimethyl 2 butanol' OR '2 [3 cyclopropylmethyl 11 hydroxy 15 methoxy 13 oxa 3 azahexacyclo [13.2.2.1 (2, 8) .0 (1, 6) .0 (6, 14) .0 (7, 12)] icosa 7, 9, 11 trien 16 yl] 3, 3 dimethyl 2 butanol' OR '21 cyclopropyl 7alpha (1 hydroxy 1, 2, 2 trimethylpropyl) 6, 14 ethano 6, 7, 8, 14 tetrahydrooripavine' OR '5 (cyclopropylmethyl) 16 [2 hydroxy 3, 3 dimethylbutan 2 yl] 15 methoxy 13 oxa 5 azahexacyclo [13.2.2.1 (2, 8) .0 (1, 6) .0 (2, 14) .0 (12, 20)] icosa 8 (20), 9, 11 trien 11 ol' OR 'acimaphin' OR 'acinorphin' OR 'addictex' OR 'addnok' OR 'ala 1000' OR 'ala1000' OR 'algesalona (buprenorphine)' OR 'anorfin' OR 'astec' OR 'belbuca' OR 'brixadi' OR 'bugnanto' OR 'bunondol' OR 'bunorfin' OR 'bunov' OR 'bupacal' OR 'bupainx' OR 'bupalster' OR 'bupan' OR 'bupeaze' OR 'bupensan' OR 'buphin' OR 'buplab' OR 'buplast' OR 'bupramyl' OR 'bupre-1 a pharma' OR 'bupre-hexal' OR 'buprefarm' OR 'buprel' OR 'bupremyl' OR 'buprenaddict' OR 'buprenal' OR 'buprenex' OR 'buprenocan' OR 'buprenoratiopharm' OR 'buprenorfin' OR 'buprenorfina' OR 'buprenorfine' OR 'buprenorphin' OR 'buprenorphine' OR 'buprenorphine hydrochloride' OR 'bupretec' OR 'buprex' OR 'buprine' OR 'busette' OR 'busiete' OR 'butec' OR 'butrans' OR 'buvera' OR 'buvidal' OR 'cam 2038' OR 'cam 2048' OR 'cam2038' OR 'cam2048' OR 'carlosafine' OR 'cl 112, 302' OR 'cl 112302' OR 'cl112, 302' OR 'cl112302' OR 'dolotec (buprenorphine)' OR 'dolotec transdermal patch' OR 'durlevatec' OR 'en 3409' OR 'en3409' OR 'espranor' OR 'eti 311' OR 'eti311' OR 'feliben' OR 'finibron' OR 'hapoctasin' OR 'indv 6200' OR 'indv6200' OR 'laribon' OR 'lepetan' OR 'lyn 013' OR 'lyn013' OR 'melodyn' OR 'mitoren' OR 'molterfin' OR 'natzon' OR 'nih 8805' OR 'nih8805' OR 'nimedol' OR 'noprex' OR 'norfinox' OR 'norphin' OR 'norspan' OR 'norvipren' OR 'panitaz' OR 'pentorel' OR 'prefibin' OR 'prefin' OR 'prekisan' OR 'prenorvine' OR 'prenotrix' OR 'probuphenine' OR 'probuphine' OR 'ramatrix' OR 'ravata' OR 'rbp 6000' OR 'rbp6000' OR 'reletrans' OR 'relevtec' OR 'rx 6029 m' OR 'rx 6029m' OR 'rx6029m' OR 'sch 028444' OR 'sch028444' OR 'sevodyne' OR 'sixmo' OR 'somnena' OR 'sovenor' OR 'sublocade' OR 'subutex' OR 'temgesic' OR 'tephine' OR 'thorbup' OR 'transtec' OR 'tranzileve' OR 'triquisic' OR 'um 952' OR 'um952')

AND

('opioid use'/exp OR 'opioid use disorder'/exp OR 'opiate addiction'/exp OR 'opiate addict' OR 'opiate addiction' OR 'opiate alkaloid addict' OR 'opiate alkaloid dependency' OR 'opiate alkaloids addict' OR 'opiate crisis' OR 'opiate dependence' OR 'opiate dependency' OR 'opiate epidemic' OR 'opioid addict' OR 'opioid addiction' OR 'opioid crisis' OR 'opioid dependence' OR 'opioid dependency' OR 'opioid epidemic' OR 'opioid-related disorders' OR 'opioids addict' OR 'opioids addiction' OR 'opioids crisis' OR 'opioids dependence' OR 'opioids dependency' OR 'opioids epidemic' OR 'opium addict' OR 'opium addiction' OR 'opium alkaloid addict' OR 'opium alkaloid addiction' OR 'opium dependence' OR 'opium dependency' OR 'opium epidemic' OR 'opiate substitution treatment'/exp OR 'opiate replacement therapy' OR 'opiate replacement treatment' OR 'opiate substitution therapy' OR 'opiate substitution treatment' OR 'opioid replacement therapy' OR 'opioid replacement treatment' OR 'opioid substitution therapy' OR 'opioid substitution treatment' OR 'heroin dependence'/exp OR 'addiction, heroin' OR 'heroin addict' OR 'heroin addiction' OR 'heroin dependence' OR 'heroin dependency' OR 'heroinism')

AND

('drug microdose'/exp OR 'drug microdosage' OR 'drug microdose' OR 'micro-dosage' OR 'micro-dose' OR 'micro-dosing' OR 'micro-dosis' OR 'microdosage' OR 'microdose' OR 'microdosing' OR 'microdosis' OR initiat*:ti,ab OR microdos*:ti,ab OR induc*:ti,ab OR bridg*:ti,ab OR replac*:ti,ab OR substitut*:ti,ab OR (low NEAR/2 dos*):ti,ab)

AND

‘human’/de

AND

[11-04-2020]/sd NOT [02-08-2024]/sd
