# Supplementary material for: Initiating buprenorphine to treat opioid use disorder without prerequisite withdrawal: an updated systematic review
Source: Addict Sci Clin Pract. 2025 Feb 20;20:19. doi: 10.1186/s13722-025-00548-z (PMC11841166; doi:10.1186/s13722-025-00548-z)
Supplement: Supplementary file 2 — Additional file 2: Study selection (Database screening flowsheet) [file 13722_2025_548_MOESM2_ESM.pptx]

## Slide 1
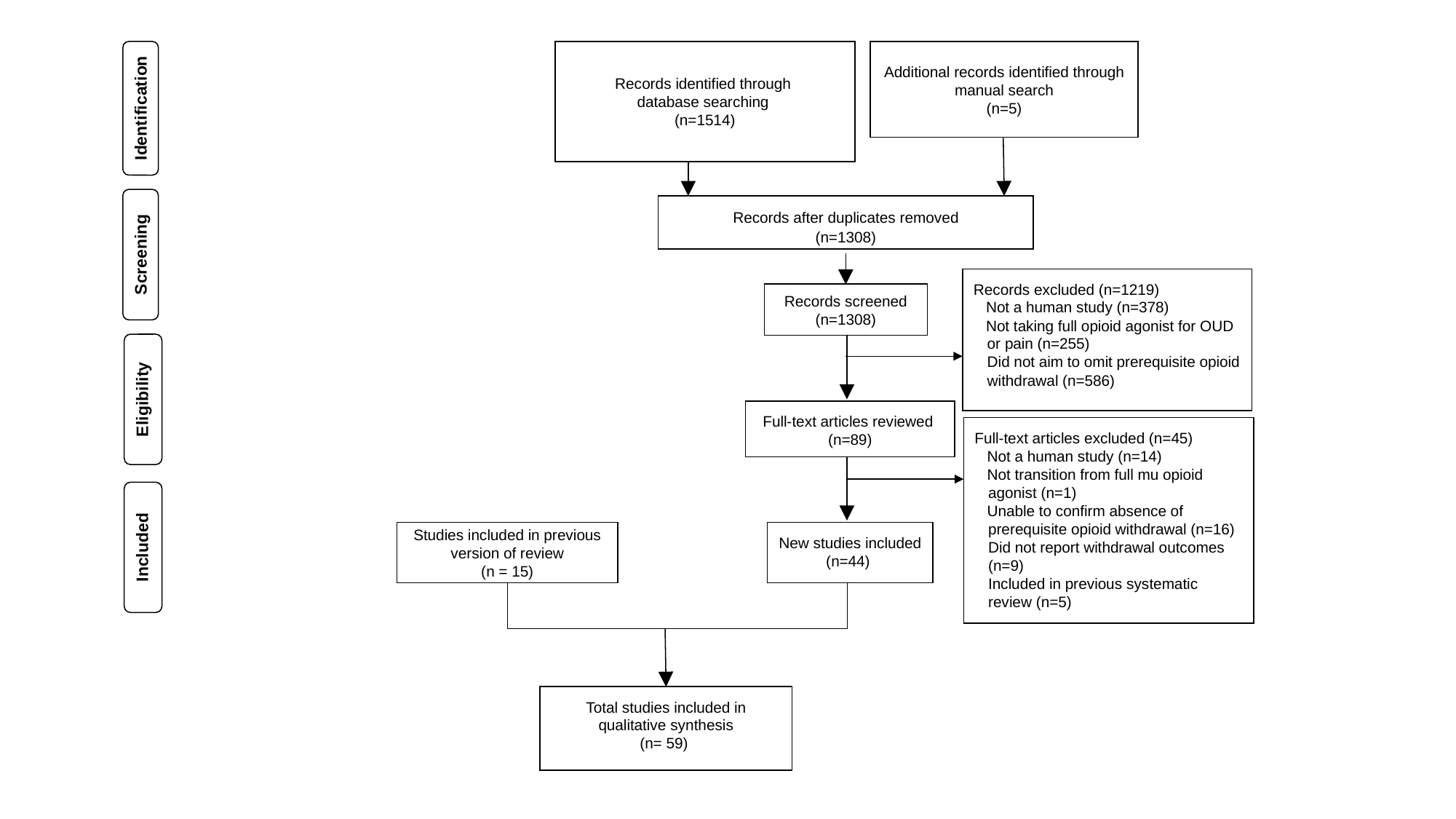

Records identified through
database searching
(n=1514)
Additional records identified through manual search
(n=5)
Identification
Records after duplicates removed
(n=1308)
Screening
Records excluded (n=1219)
 Not a human study (n=378)
 Not taking full opioid agonist for OUD or pain (n=255)
Did not aim to omit prerequisite opioid withdrawal (n=586)
Records screened
(n=1308)
Eligibility
Full-text articles reviewed
(n=89)
Full-text articles excluded (n=45)
 Not a human study (n=14)
 Not transition from full mu opioid agonist (n=1)
 Unable to confirm absence of prerequisite opioid withdrawal (n=16)
Did not report withdrawal outcomes (n=9)
Included in previous systematic review (n=5)
Studies included in previous version of review
(n = 15)
New studies included (n=44)
Included
Total studies included in qualitative synthesis
(n= 59)
