# Supplementary material for: Initiating buprenorphine to treat opioid use disorder without prerequisite withdrawal: an updated systematic review
Source: Addict Sci Clin Pract. 2025 Feb 20;20:19. doi: 10.1186/s13722-025-00548-z (PMC11841166; doi:10.1186/s13722-025-00548-z)
Supplement: Supplementary file 3 — Additional file 3: Tables (Table 1: Patient characteristics in case reports/case series. Table 2: Summary of dosing strategy characteristics from case reports/case series. Table 3: Summary of patient characteristics from single-arm observational studies. Table 4: Summary of dosing strategies from single-arm observational studies Appendix Table 1: Risk of bias summary of case reports/case series. Appendix Table 2: Risk of bias summary of single-arm observational studies. Appendix Table 3: Additional case report/observational study references. Appendix Table 4: Case reports/case series summary table. Appendix Table 5: Single arm observational studies summary table.) [file 13722_2025_548_MOESM3_ESM.docx]

Table 1. Patient Characteristics in Case Reports/Case Series

| Feature | N (%) |
| --- | --- |
| Patient cases | 106 |
| Country  Australia  Canada  Slovenia  Switzerland  United Kingdom  United States of America | 1  14 1  2  1  27 |
| Female | 48 (45)* |
| Age (median, range) | 40 (16 – 73) |
| Indication for buprenorphine  OUD  Pain  Both | 63 (59)  17 (16)  26 (25) |
| Initiation Setting  Inpatient  Outpatient  Inpatient and outpatient | 39 (37)  63 (59)  4 (4) |
| Initiation Strategy  Buccal  Intravenous  Patch  Sublingual | 8 (7)  3 (3)  17 (16)  78 (74) |
| Abbreviations: LDBI = low dose buprenorphine initiation; OUD=opioid use disorder  * Not reported for two patients | |

Table 2. Summary of dosing strategy characteristics from case reports/case series

| Initiation Strategy | No. of patients* | History of heroin or fentanyl use n (%) | Opioid regimen immediately prior to initiation, n (%)^†^ | Initial buprenorphine dose (median, range) | Time of buprenorphine and full opioid agonist overlap (median, range)^§^ | Time to complete initiation, median (range) ^§^ | Any withdrawal during initiation, n (%) | Moderate or severe withdrawal during initiation, n (%) | Validated scale used to measure withdrawal | Transition to buprenorphine monotherapy, n (%) |
| --- | --- | --- | --- | --- | --- | --- | --- | --- | --- | --- |
| Buccal | 8 | 7 (88) | 5 (63) methadone  4 (50) short acting opioids  1 (13) fentanyl infusion | 225 (225 – 300) ug | 7 (4 – 11) days | 9 (6 – 11) days | 8 (100) | 1 (13) | 7 (88) | 8 (100) |
| Intravenous | 3 | 1 (33) | 2 (66) methadone  1 (33) short acting opioids | 0.15 (0.1 – 0.15) mg | 4 (4 – 25) days | 6 (5 – 25) days | 1 (33) | 0 (0) | 1 (33) | 3 (100) |
| Patch | 17 | 11 (65) | 6 (35) fentanyl/heroin  2 (12) long acting opioids  9 (53) methadone  1 (6) NR  7 (41) short acting opioids | 20 (5 – 120) ug/hr | 5 (1 – 14) days | 10 (4 – 16) days | 13 (76) | 3 (18) | 6 (35) | 13 (76) |
| Sublingual | 78 | 43 (55) | 18 (23) fentanyl/heroin  6 (8) fentanyl infusion  14 (18) long acting opioids  29 (37) methadone  2 (3) NR  25 (32) short acting opioids | 0.5 (0.15 – 1) mg | 7 (1 – 120) days | 8 (3 – 120) days | 35 (45) | 9 (12) | 24 (31) | 56 (72)^¶^ |
| Abbreviations: hr=hour; LDBI = low dose buprenorphine initiation; mg = milligram; NR = not reported; ug = microgram  *Cases that did not provide adequate information to determine if the listed outcome occurred were not included  ^†^ Categories are not mutually exclusive  ^§^ Patients who did not complete initiation or if information was not reported are not included  ^¶^ In three additional patients: one patient reported relapse and did not return for subsequent subcutaneous dose; The second patient required 3 attempts to transition; The third patient expired in the intensive care unit prior to completing the regimen | | | | | | | | | | |

Table 3: Summary of Patient Characteristics from Single-Arm Observational Studies

| Study author | Country | No. of patients | Median Age* | Male, N (%) | Indication, N (%) | Treatment Setting (inpatient vs. outpatient) | History of heroin or fentanyl use, N (%)^†^ | Opioid regimen immediately prior to initiation^†^ |
| --- | --- | --- | --- | --- | --- | --- | --- | --- |
| Adams | USA | 45 | 48 | 25 (56) | 18 (40) Both  22 (49) OUD  5 (11) Pain | Inpatient | 36 (80) | 16 (36) short acting opioids  25 (56) methadone  1 (2) long-acting opioids  3 (7) fentanyl |
| Arnouk | USA | 42 (46 initiations) | 48 | 31 (74) | 14 (33) Both  24 (57) OUD  4 (10) Pain | Inpatient | 31 (74) | Median MME 150 (73-208) |
| Bhatraju | USA | 62 | 44 | 37 (60) | 62 (100) OUD | Inpatient | NR | 42 (68) methadone  9 (15) fentanyl  62 (100) short-acting opioids  Median MME 228 (12-1505) |
| Hayes | USA | 28 | 45 for unsuccessful initiations; 53 for successful initiations | 16 (57) | 6 (21) Both  16 (57) OUD  6 (21) Pain | Inpatient | 13 (46) | 17 (61) methadone |
| Jablonski | USA | 59 | 53 | 25 (42) | 47 (80) Both  12 (20) OUD | Inpatient | 42 (71) | 8 (14) chronic opioid prescription |
| Jones | USA | 126 (175 initiations) | 37* | 90 (71)^††^ | 33 (26) Both  93 (74) OUD | Outpatient | 126 (100) | 126 (100) fentanyl |
| Murray | USA | 33 | 58 | 20 (61) | 33 (100) Both | Inpatient | NR; 22 (67) + for opiates on admission | 7 (21) methadone  Median MME 30.8 (15-60) |
| Naren | Australia | 32 | NR (Range 28 – 69) | 19 (59) | 32 (100) OUD | Outpatient | 7 (22) | 32 (100) methadone |
| Noel | USA | 27 | 43 | 17 (63) | 9 (33) Both  18 (67) OUD | Outpatient | 27 (100) | 27 (100) fentanyl |
| Raheemullah | USA | 15 | 47 | 11 (73) | 15 (100) Both | Inpatient | NR | Median MME 90 (30-341) |
| Schult | USA | 71  (76 initiations) | 39* | 48 (68) | 59 (83) OUD  12 (17) OD | Inpatient | NR | Median MME 1000 |
| Sokolski | USA | 24 | 41* | 14 (58) | 24 (100) OUD | Inpatient | 13 (54) | Mean MME 171 (19-428) |
| Suen | USA | 12 | 34 | 8 (67) | 3 (25) Both  9 (75) OUD | Outpatient | 11 (92) fentanyl  8 (67) heroin | NR |
| MME = morphine milliequivalent; NR = not reported; OUD = opioid use disorder; OD = opioid dependence; USA = United States of America  ^*^ Indicates mean was reported instead of median  ^†^ Categories are not mutually exclusive. If opioid categories were not reported, MME or alternative information was reported if available ^††^ 3 additional patients reported as non-binary | | | | | | | | |

Table 4: Summary of Dosing Strategies from Single-Arm Observational Studies

| Study author | Initial buprenorphine dose described in protocol | No. of patients | Protocol description | How was withdrawal assessed | Withdrawal outcomes | Transition to buprenorphine ^Ω^ |
| --- | --- | --- | --- | --- | --- | --- |
| Buccal (ug) | | | | | | |
| Adams | 225 ug^*^ | 45 | 7-day protocol with 6 days of full opioid agonist overlap  38 completed in < 7 days  5 completed in >7 days  2 completion time NR^*^ | COWS^†^ | Median max COWS during initiation 7 (1-18)  6/24 (25%) with COWS scoring experienced moderate withdrawal | 36/45 (80%) transitioned to buprenorphine |
| Arnouk | 150 ug | 42 (46 initiations) | Short protocol: 5-day protocol with 3 days of full opioid agonist overlap. Time to complete (median, IQR): 4 (3-5) days  Long protocol: 8-day protocol with 6 days of full opioid agonist overlap. Time to complete (median, IQR): 7 (6-9) days | Progress note review | 15/46 (33%) attempts  experienced withdrawal; “majority described as mild events”  2/46 (4%) withdrawal severe enough to stop initiation | 36/46 (78%) attempts resulted in transition to buprenorphine |
| Hayes | 225 ug^μ^ | 28 | Short-acting opioids protocol: 4-day protocol with 3 days of full opioid agonist overlap  Long-acting opioids protocol: 7-day protocol with 6 days of full opioid agonist overlap  Successful initiation attempts lasted a median of 6 days (IQR range 4 - 7 days)  Unsuccessful initiation attempts lasted a median of 3 days (IQR range 2 -6 days) | COWS & reports of precipitated withdrawal | Median maximum COWS for successful initiation: 1 (IQR 0-2)  Median maximum COWS for unsuccessful initiation: 5 (IQR 1-12)  7/28 (25%) experienced COWS score >7  4/28 (14%) experienced precipitated withdrawal | 19/28 (68%) transitioned to buprenorphine |
| Intravenous (mg) | | | | | | |
| Jablonski | 0.15 or 0.3 mg | 59 | Dosing strategy provided for initial 24-48 hrs. After reaching dose of 4 mg SL buprenorphine, dosing was at discretion of provider  Median time to achieve dose of 16 mg SL was 65 hours (IQR 56-72 hours) | COWS | 28/59 (47%) minimal withdrawal  27/59 (46%) mild withdrawal  4/59 (7%) moderate withdrawal | 54/59 (92%) transitioned to buprenorphine |
| Murray | 0.15 mg | 33 | Two protocols (standard and slow regimens) over 5 days. Full opioid agonists were continued throughout and beyond the buprenorphine titration process as “clinically appropriate” | COWS | Mean COWS scores did not exceed 3.5  1/33 (3%) discontinued due to pain and withdrawal  3/33 (9%) had regimen adjusted due to uncontrolled pain | 30/33 (91%) transitioned to buprenorphine  19/30 (63%) transitioned to buprenorphine monotherapy  11/30 (37%) transitioned to buprenorphine with concurrent full opioid agonist(s) |
| Patch (ug/hr) | | | | | | |
| Naren | 10 ug/hr (32, 100%) | 32 | 28-day protocol of methadone and buprenorphine patch overlap with flexibility between days 26 – 28 to cease full opioid agonists | Patient reported withdrawal | 12/32 (38%) no withdrawal  9/32 (28%) mild withdrawal  3/32 (9%) withdrawal; severity not specified  4/32 (13%) withdrawal severe or leading to buprenorphine cessation  4/32 (13%) withdrawal data not reported | 23/32 (72%) transitioned to buprenorphine |
| Raheemullah | 10 ug/hr (2, 13%)  20 ug/hr (13 (87%) | 15 | Patient-specific protocols that were all completed within 4 days and included 1 – 3 days of full opioid agonist overlap | COWS | 15/15 (100%) experienced mild or minimal withdrawal | 15/15 (100%) transitioned to buprenorphine  12/15 (80%) transitioned to buprenorphine monotherapy  3/15 (20%) transitioned to buprenorphine with concurrent full opioid agonist(s) |
| Sokolski | 20 ug/hr | 24 | 4-day protocol up to 9 mg with at least 3 days of full opioid agonist overlap. After 9mg, additional adjustments of buprenorphine and full opioid agonists occurred as needed.  Initiation was considered complete at first dose of 8 mg SL, which occurred at a mean of 72 (56 – 90) hours. | Precipitated withdrawal | 1/24 (4%) discontinued due to persistent withdrawal | 19/24 (79%) transitioned to 8 mg or more of buprenorphine |
| Sublingual (mg) | | | | | | |
| Bhatraju | 0.5 mg | 62 | 8-day protocol with7 days of full opioid agonist overlap.  Patients completed buprenorphine initiation over a mean of 8 (2 – 35) days. | Progress note review | 23/62 (37%) experienced any withdrawal; most described as minor | 51/62 (82%) transitioned to buprenorphine  28/51 (55%) transitioned to buprenorphine monotherapy  23/51 (45%) transitioned to buprenorphine with concurrent full opioid agonist(s) |
| Jones | 0.5 mg | 126 (175 initiations) | Shared decision making among provider and patient to choose a 4 or 7-day protocol  Patients were counseled on the process and common concerns including instructions to continue full-agonist opioids as much as needed to avoid withdrawal symptoms. Patients were instructed to follow up with a provider once they had achieved a buprenorphine dose of 12 mg. | Components of SOWS scores, precipitated withdrawal, progress note review | Note: 118/175 initiation attempts (67%) had recorded withdrawal data  81/118 (69%) initiation attempts experienced no withdrawal  25/118 (21%) attempts experienced mild withdrawal  10/118 (8%) attempts experienced moderate withdrawal  2/118 (2%) attempts experienced severe withdrawal  10/118 (8%) attempts experienced precipitated withdrawal; 7 of these 10 were a result of protocol deviations  86/118 (73%) attempts did not experience precipitated withdrawal  22/118 (19%) attempts study authors could not determine if precipitated withdrawal occurred | NR – outcomes reported in initiation attempts; did not report transition rates |
| Noel | 0.5 mg | 27 | 7-day protocol of buprenorphine titration; after day 7 was at discretion of the clinician.  Patients were informed continuation of full opioid agonists during initiation is common to avoid withdrawal until the buprenorphine has increased to a therapeutic dose (8 mg) | Patient reported symptoms | 3/14 (21%) patients who completed protocol experienced mild withdrawal  3/7 (43%) patient who did not complete protocol discontinued due to withdrawal | 14/27 (52%) completed protocol and transitioned to buprenorphine  4/14 (29%) transitioned to buprenorphine monotherapy  10/14 (71%) transitioned to buprenorphine with concurrent full opioid agonist(s) |
| Schult | 0.15 mg | 71  (76 initiations) | 5-day protocol which was completed over a median of 5 days (range 3 – 6 days; IQR 5 – 5 days) in the 54 patients who completed initiation | Precipitated withdrawal | 2/71 (3%) experienced precipitated withdrawal | 54/71 (76%) reached dose of 4 mg SL buprenorphine; some required multiple attempts  Median MME of full opioid agonist received 24h after reaching target buprenorphine dose: 37.5 mg; number of patients receiving concurrent full opioid agonist(s) NR |
| Suen | 0.5 mg | 12 | 4-day protocol with 3 days of full agonist overlap with subsequent adjustments | Patient reported symptoms | 1/12 (8%) mild withdrawal  6/12 (50%) no withdrawal | 7/12 (58%) transitioned to buprenorphine |
| Abbreviations: COWS = Clinical Opiate Withdrawal Scale; hr = hour; IQR= interquartile range; mg = milligram; MME = morphine milliequivalent; NR = not reported; SL = sublingual; ug = microgram  ^*^ 2/45 had augmentation with buprenorphine patch; Dose of patch NR; Time to complete initiation NR  ^†^ Only 24/45 had consistently documented COWS scores  ^Ω^Number continuing on concurrent full opioid agonist(s) not always explicitly stated  ^μ^ 3 patients received a 20 ug/hr patch prior to starting buccal buprenorphine | | | | | | |

Appendix Table 1. Risk of Bias Summary of Case Reports/Case Series

| Author, year | Clear selection methods | Adequately ascertained exposure | Outcome adequately ascertained | Alternative causes of the observation ruled out | Follow-up long enough | Sufficient detail to replicate |
| --- | --- | --- | --- | --- | --- | --- |
| Ahmed, 2023 | N | N | N | N | Y | Y |
| Anderson, 2023 | N | Y | Y | N | Y | Y |
| Azar, 2020 | N | Y | Y | N | Y | Y |
| Azar, 2023 | N | Y | Y | N | Y | Y |
| Azar, 2024.1 | N | Y | Y | N | Y | Y |
| Azar, 2024.2 | N | Y | Y | N | Y | Y |
| Becker, 2020 | Y | N | N | N | Y | N |
| Brar, 2020 | N | N | N | N | Y | Y |
| Buchheit, 2021 | N | N | N | N | Y | Y |
| Cassells, 2021 | N | N | N | N | Y | N |
| Crane, 2021 | N | Y | Y | N | Y | Y |
| Hailozian, 2022 | N | N | Y | N | Y | Y |
| James, 2021 | N | Y | N | N | Y | Y |
| Junn, 2023 | Y | N | N | N | Y | N |
| Kaliamurthy, 2023 | N | N | N | N | Y | Y |
| Kinasz, 2020 | N | Y | Y | N | Y | N |
| Lavre, 2024 | N | N | N | N | Y | Y |
| Leyde, 2021 | N | Y | Y | N | Y | Y |
| Martell, 2022 | N | Y | Y | N | Y | Y |
| Menard, 2021 | N | Y | N | N | Y | Y |
| Menard 2022 | N | N | N | N | Y | N |
| Mortaji, 2021 | N | Y | N | N | Y | Y |
| Patel, 2024 | N | N | N | N | Y | Y |
| Reddy, 2024 | N | N | Y | N | Y | N |
| Robbins, 2020 | Y | N | N | N | Y | Y |
| Szczesniak, 2024 | N | Y | Y | N | Y | Y |
| Seval, 2023 | Y | Y | Y | N | Y | Y |
| Shelton, 2024 | N | Y | Y | N | Y | Y |
| Singh, 2021 | N | Y | Y | N | Y | Y |
| Thakar, 2022 | N | Y | N | N | Y | Y |
| Vytialingam, 2021 | N | Y | Y | N | Y | Y |

Appendix Table 2. Risk of Bias Summary of Single Arm Observational Studies

| Author, year | Clearly stated aim | Inclusion of consecutive patients | Prospective collection of data | Endpoint appropriate to aim of the study | Unbiased assessment of study endpoint | Follow up period appropriate to aim of the study | Loss to follow up less than 5% | Prospective calculation of study size | Total Score |
| --- | --- | --- | --- | --- | --- | --- | --- | --- | --- |
| Adams, 2023 | 1 | 2 | 2 | 1 | 1 | 2 | 2 | 0 | 11 |
| Arnouk, 2023 | 2 | 2 | 2 | 1 | 1 | 2 | 2 | 0 | 12 |
| Bhatraju, 2022 | 2 | 2 | 2 | 1 | 1 | 1 | 2 | 0 | 11 |
| Hayes, 2024 | 2 | 2 | 2 | 1 | 1 | 2 | 2 | 0 | 12 |
| Jablonski, 2022 | 2 | 1 | 1 | 2 | 1 | 2 | 2 | 0 | 11 |
| Jones, 2024 | 2 | 2 | 2 | 1 | 2 | 2 | 0 | 0 | 11 |
| Murray 2023 | 0 | 2 | 2 | 2 | 1 | 2 | 2 | 0 | 11 |
| Naren, 2024 | 1 | 2 | 2 | 1 | 1 | 2 | 2 | 0 | 11 |
| Noel, 2023 | 2 | 1 | 1 | 1 | 1 | 2 | 1 | 0 | 9 |
| Raheemullah, 2021 | 1 | 2 | 2 | 2 | 1 | 2 | 2 | 0 | 12 |
| Schult, 2023 | 2 | 2 | 2 | 2 | 1 | 1 | 2 | 0 | 12 |
| Sokolski, 2023 | 2 | 2 | 2 | 2 | 1 | 2 | 2 | 0 | 13 |
| Suen, 2022 | 1 | 1 | 2 | 1 | 1 | 2 | 1 | 0 | 9 |
| Methodological index for non-randomized studies (MINORS) tool: 0= not reported; 1= reported but inadequate; 2= reported and adequate | | | | | | | | | |

Appendix Table 3. Additional Case Report/Observational Study References

| **First Author Last Name, year** | **Full Citation** |
| --- | --- |
| **Case Reports/Case Series** | |
| Ahmed, 2023 | Ahmed S, Faruqui Z, Poddar K, et al. Low-dose buprenorphine initiation in the era of fentanyl and fentanyl analogs: A case series of outpatient inductions. *J Opioid Manag.* 2023;19(5):455-60 |
| Anderson, 2023 | Anderson C, Cooley R, Patil D. Transitioning from high-dose methadone to buprenorphine using a microdosing approach: Unique considerations at ASAM Level 3 facilities. *J Addict Med.* 2023;17(2):241-4 |
| Azar, 2020 | Azar P, Wong JSH, Jassemi S, et al. A case report: Rapid micro-induction of buprenorphine/naloxone to administer buprenorphine extended-release in an adolescent with severe opioid use disorder. *Am J Addict.* 2020;29(6):531-5 |
| Azar, 2023 | Azar P, Mathew N, Mahal D, et al. Developing a rapid transfer from opioid full agonist to buprenorphine: “Ultrarapid micro-dosing” proof of concept. *J Psychoactive Drugs.* 2023;55(1):94-101 |
| Azar, 2024.1 | Azar P, Schneiderman H, Barron H, et al. Rapid induction of transdermal buprenorphine to subcutaneous extended-release buprenorphine for the treatment of opioid use disorder. *Addict Sci Clin Pract*. 2024;19(1):50. |
| Azar, 2024.2 | Azar P, Wong JSH, Mathew N, et al. 48-hour induction of transdermal buprenorphine to extended-release buprenorphine. *J Addict Med*. 2024;18(1):82-85. |
| Becker, 2020 | Becker WC, Frank JW, Edens EL. Switching from high-dose, long-term opioids to buprenorphine: A case series. *Ann Intern Med.* 2020;173(1):70-1 |
| Brar, 2020 | Brar R, Fairbairn N, Sutherland C, et al. Use of a novel prescribing approach for the treatment of opioid use disorder: Buprenorphine/naloxone micro-dosing- a case report. *Drug Alcohol Rev.* 2020;39(5):588-94 |
| Buchheit, 2021 | Buchheit BM, Joslin T, Turner HN, et al. Ambulatory microdose induction of buprenorphine-naloxone in two adolescent patients with sickle cell disease. *Pediatr Blood Cancer.* 2021;68(1):e28766 |
| Cassells, 2021 | Cassells N, Hill DR, Marr E, et al. Microdosing case study series-transferring from methadone to oral buprenorphine. *Heroin Addict Relat Clin Probl.* 2021;23(5):57-66 |
| Crane, 2021 | Crane K, Snead J, Stanley R, et al. Intravenous buprenorphine micro-dosing induction in a patient on methadone treatment: A case report. *J Acad Consult Liaison Psychiatry.* 2021;62(2):243-7 |
| Hailozian, 2022 | Hailozian C, Luftig J, Liang A, et al. Synergistic effect of ketamine and buprenorphine observed in the treatment of buprenorphine precipitated opioid withdrawal in a patient with fentanyl use. *J Addict Med.* 2022;16(4):483-7 |
| James, 2021 | James H, Nolan S, Fairbairn N. Rapid induction of buprenorphine/naloxone from methadone using a micro-dosing approach for opioid use disorder treatment in an inpatient setting: A case report. *UBCMJ.* 2021;13(1):23-5 |
| Junn, 2023 | Junn S, Tugarinov N, Mark K. Low-dose induction of buprenorphine in pregnancy: A case series. *J Addict Med.* 2024;18(1):62-4 |
| Kaliamurthy, 2023 | Kaliamurthy S, Jegede O, Hermes G. Community based buprenorphine micro-induction in the context of methadone maintenance treatment and fentanyl-Case report. *J Addict Dis.* 2023;41(2):175-80 |
| Kinasz, 2020 | Kinasz KR, Herbst ED, Kalapatapu RK. Case report: Buprenorphine induction using transdermal buprenorphine in a Veteran with opioid use disorder and psychosis, managing precipitated withdrawal. *Mil Med.* 2020;185(9-10):e1872-e1875 |
| Lavre, 2024 | Micro-dosing with buprenorphine when switching from full agonists (methadone, SR-morphine) to buprenorphine treatment in opioid  agonist-assisted treatment programmes in Slovenia*. Heroin Addict Relat Clin Probl 2024; 26, 3* https://doi.org/10.62401/2531-4122-2024-3 |
| Leyde, 2021 | Leyde S, Suen L, Pratt L, et al. Transition from oxycodone to buprenorphine/naloxone in a hospitalized patient with sickle cell disease: A case report. *J Gen Intern Med.* 2022;37(5):1281-5 |
| Martell, 2022 | Martell JP, Konakanchi JS, Sethi R. Treating opioid use disorder with rapid micro induction technique of sublingual buprenorphine/naloxone in an outpatient setting-a case report. *J Addict Dis.* 2022;40(3):439-43 |
| Menard, 2021 | Menard S, Jhawar A. Microdose induction of buprenorphine-naloxone in a patient using high dose methadone: A case report. *Ment Health Clin.* 2021;11(6):369-782 |
| Menard 2022 | Menard S, Jhawar A. Outpatient microdose induction with transdermal buprenorphine: A case series. *Healthcare (Basel).* 2022;10(7):1307 |
| Mortaji, 2021 | Mortaji P, Terasaki D, Moo-Young J. Advanced inpatient management of opioid use disorder in a patient requiring serial surgeries. *J Gen Intern Med.* 2021;36(8):2448-51 |
| Patel, 2024 | Patel NB, Parilla BV. Buprenorphine induction using microdosing for the management of opioid use disorder in pregnancy. *AJP Rep*. 2024;14(1):e88-e90. |
| Reddy, 2024 | Reddy S, Martin CE. Low-dose buprenorphine initiation during pregnancy: A case report. *AJOG Glob Rep.* 2024;4(1):100308 |
| Robbins, 2020 | Robbins JL, Englander H, Gregg J. Buprenorphine microdose induction for the management of prescription opioid dependence. *J Am Board Fam Med.* 2021;34(Suppl):S141-6 |
| Szczesniak, 2024 | Szczesniak, L., Britton, S., RN, T.B. *et al.* Low-dose overlap initiation with split tablets of buprenorphine in intubated intensive care unit patients with opioid use disorder. *Harm Reduct J* **21**, 114 (2024). https://doi.org/10.1186/s12954-024-01028-4 |
| Seval, 2023 | Seval N, Nunez J, Roth P, et al. Inpatient low-dose transitions from full agonist opioids including methadone onto long-acting depot buprenorphine: Case series from a multicenter clinical trial. *J Addict Med.* 2023;17(4):e232-239 |
| Shelton, 2024 | Shelton T, Nama S, Hall O, Williams M. Case report: Successful induction of buprenorphine in medically complex patients concurrently on opioids: a case series at a tertiary care center. *Front Pharmacol*. 2024;15:1335345. |
| Singh, 2021 | Singh G, Konakanchi JS, Betsch B, et al. Rapid microinduction of sublingual buprenorphine from methadone in an outpatient setting: “A case series.” *J Opioid Manag.* 2021;17(7):167-70 |
| Thakar, 2022 | Thakrar AP, Jablonski L, Ratner J, et al. Micro-dosing intravenous buprenorphine to rapidly transition from full opioid agonists. *J Addict Med.* 2022;16(1):122-4 |
| Vytialingam, 2021 | Vytialingam RC, Schug SA, O’Regan R. Successful rotation from long-acting full agonist opioids to sublingual buprenorphine/naloxone using a microdosing approach. *J Opioid Manag.* 2021;17(7):159-66 |
| **Single Arm Observational Studies** | |
| Adams, 2023 | Adams KK, Cohen SM, Guerra ME, et al. Low-dose initiation of buprenorphine in hospitalized patients using buccal buprenorphine: A case series. *J Addict Med.* 2023;17(4):474-6 |
| Arnouk, 2023 | Arnouk S, Wunderlich JR, Sidelnik SA. Evaluation of low-dose buprenorphine initiation with buprenorphine buccal films in hospitalized patients: A retrospective cohort study. *J Addict Med.* 2024;18(1):42-7 |
| Bhatraju, 2022 | Bhatraju EP, Klein JW, Hall AN, et al. Low dose buprenorphine induction with full agonist overlap in hospitalized patients with opioid use disorder: A retrospective cohort study. *J Addict Med.* 2022;16(4):461-5 |
| Hayes, 2024 | Hayes BT, Li P, Nienaltow T, et al. Low-dose buprenorphine initiation and treatment continuation among hospitalized patients with opioid dependence: A retrospective cohort study. *J Subst Use Addict Treat.* 2024;158:209261 |
| Jablonski, 2022 | Jablonski LA, Bodnar AR, Stewart RW. Development of an intravenous low-dose buprenorphine initiation protocol. *Drug Alcohol Depend.* 2022;237:109541 |
| Jones, 2024 | Jones BLH, Geier M, Neuhaus J, et al. Withdrawal during outpatient low dose buprenorphine initiation in people who use fentanyl: a retrospective cohort study. *Harm Reduct J*. 2024;21(1):80. |
| Murray 2023 | Murray JP, Pucci G, Weyer G, et al. Low dose IV buprenorphine inductions for patients with opioid use disorder and concurrent pain: A retrospective case series. *Addict Sci Clin Pract.* 2023;18(1):38 |
| Naren, 2024 | Naren T, Cook J, MacCartney P, Membrey D. Buprenorphine microdosing regimen using transdermal buprenorphine patches to transition from methadone to buprenorphine. *Drug Alcohol Rev*. 2024;43(4):1013-1018. |
| Noel, 2023 | Noel M, Abbs E, Suen L, et al. The Howard Street method: A community pharmacy=led low dose overlap buprenorphine initiation protocol for individuals using fentanyl. *J Addict Med.* 2023;17(4):e255-e261 |
| Raheemullah, 2021 | Raheemullah A, Lembke A. Buprenorphine induction without opioid withdrawal: A case series of 15 opioid-dependent inpatients induced on buprenorphine using microdoses of transdermal buprenorphine. *Am J Ther.* 2019;28(4):e504-e508 |
| Schult, 2023 | Schult RF, Maynard KM, Corvelli JM, et al. Low-dose initiation of buprenorphine in hospitalized patients on full agonist opioid therapy: A retrospective observational study. *J Addict Med.* 2023;17(6):685-90 |
| Sokolski, 2023 | Sokolski E, Skogrand E, Goff A, et al. Rapid low-dose buprenorphine initiation for hospitalized patients with opioid use disorder. *J Addict Med.* 2023;17(4):e278-80 |
| Suen, 2022 | Suen LW, Lee TG, Silva M, et al. Rapid overlap initiation protocol using low dose buprenorphine for opioid use disorder treatment in an outpatient setting: A case series. *J Addict Med.* 2022;16(5):534-40 |

Appendix Table 4: Case Reports/Case Series Summary Table

| Dosage form | No. of patients* | History of heroin or fentanyl use n (%) | Initial buprenorphine dose (median, range) | Time of buprenorphine and full opioid agonist overlap (median, range)^§^ | Time to complete initiation, median (range) ^§^ | Withdrawal outcomes |
| --- | --- | --- | --- | --- | --- | --- |
| Buccal | 8 | 7 (88) | 225 (225 – 300) ug | 7 (4 – 11) days | 9 (6 – 11) days | 8 (100%) experienced any withdrawal  1 (13%) experienced moderate or severe withdrawal |
| Intravenous | 3 | 1 (33) | 0.15 (0.1 – 0.15) mg | 4 (4 – 25) days | 6 (5 – 25) days | 1 (33%) experienced any withdrawal  0 (0%) experienced moderate or severe withdrawal |
| Patch | 17 | 11 (65) | 20 (5 – 120) ug/hr | 5 (1 – 14) days | 10 (4 – 16) days | 13 (76%) experienced any withdrawal   3 (18%) experienced moderate or severe withdrawal |
| Sublingual | 78 | 43 (55) | 0.5 (0.15 – 1) mg | 7 (1 – 120) days | 8 (3 – 120) days | 35 (45%) experienced any withdrawal   9 (12%) experienced moderate or severe withdrawal |
| Abbreviations: hr=hour; LDBI = low dose buprenorphine initiation; mg = milligram; NR = not reported; ug = microgram  *Cases that did not provide adequate information to determine if the listed outcome occurred were not included  ^§^ Patients who did not complete initiation or if information was not reported are not included | | | | | | |

Appendix Table 5: Single Arm Observational Studies Summary Table

| Study author | Treatment setting of patient population | Dosage Form | Dosing details | Withdrawal outcomes |
| --- | --- | --- | --- | --- |
| Adams | Inpatient | Buccal | 7-day protocol with 6 days of full opioid agonist overlap  38 completed in < 7 days  5 completed in >7 days  2 completion time NR^*^ | Median max COWS during initiation 7 (1-18)  6/24 (25%) with COWS scoring experienced moderate withdrawal |
| Arnouk | Inpatient | Buccal | Short protocol: 5-day protocol with 3 days of full opioid agonist overlap. Time to complete (median, IQR): 4 (3-5) days  Long protocol: 8-day protocol with 6 days of full opioid agonist overlap. Time to complete (median, IQR): 7 (6-9) days | 15/46 (33%) attempts  experienced withdrawal; “majority described as mild events”  2/46 (4%) withdrawal severe enough to stop initiation |
| Bhatraju | Inpatient | Sublingual | 8-day protocol with7 days of full opioid agonist overlap.  Patients completed buprenorphine initiation over a mean of 8 (2 – 35) days. | 23/62 (37%) experienced any withdrawal; most described as minor concurrent full opioid agonist(s) |
| Hayes | Inpatient | Buccal | Short-acting opioids protocol: 4-day protocol with 3 days of full opioid agonist overlap  Long-acting opioids protocol: 7-day protocol with 6 days of full opioid agonist overlap  Successful initiation attempts lasted a median of 6 days (IQR range 4 - 7 days)  Unsuccessful initiation attempts lasted a median of 3 days (IQR range 2 -6 days) | Median maximum COWS for successful initiation: 1 (IQR 0-2)  Median maximum COWS for unsuccessful initiation: 5 (IQR 1-12)  7/28 (25%) experienced COWS score >7  4/28 (14%) experienced precipitated withdrawal |
| Jablonski | Inpatient | Intravenous | Dosing strategy provided for initial 24-48 hrs. After reaching dose of 4 mg SL buprenorphine, dosing was at discretion of provider  Median time to achieve dose of 16 mg SL was 65 hours (IQR 56-72 hours) | 28/59 (47%) minimal withdrawal  27/59 (46%) mild withdrawal  4/59 (7%) moderate withdrawal |
| Jones | Outpatient | Sublingual | Shared decision making among provider and patient to choose a 4 or 7-day protocol  Patients were counseled on the process and common concerns including instructions to continue full-agonist opioids as much as needed to avoid withdrawal symptoms. Patients were instructed to follow up with a provider once they had achieved a buprenorphine dose of 12 mg. | 81/118 (69%) initiation attempts experienced no withdrawal  25/118 (21%) attempts experienced mild withdrawal  10/118 (8%) attempts experienced moderate withdrawal  2/118 (2%) attempts experienced severe withdrawal  10/118 (8%) attempts experienced precipitated withdrawal; 7 of these 10 were a result of protocol deviations  86/118 (73%) attempts did not experience precipitated withdrawal  22/118 (19%) attempts study authors could not determine if precipitated withdrawal occurred  Note: 118 of attempts had recorded withdrawal data |
| Murray | Inpatient | Intravenous | Two protocols (standard and slow regimens) over 5 days. Full opioid agonists were continued throughout and beyond the buprenorphine titration process as “clinically appropriate” | Mean COWS scores did not exceed 3.5  1/33 (3%) discontinued due to pain and withdrawal  3/33 (9%) had regimen adjusted due to uncontrolled pain |
| Naren | Outpatient | Patch | 28-day protocol of methadone and buprenorphine patch overlap with flexibility between days 26 – 28 to cease full opioid agonists | 12/32 (38%) no withdrawal  9/32 (28%) mild withdrawal  3/32 (9%) withdrawal; severity not specified  4/32 (13%) withdrawal severe or leading to buprenorphine cessation  4/32 (13%) withdrawal data not reported |
| Noel | Outpatient | Sublingual | 7-day protocol of buprenorphine titration; after day 7 was at discretion of the clinician.  Patients were informed continuation of full opioid agonists during initiation is common to avoid withdrawal until the buprenorphine has increased to a therapeutic dose (8 mg) | 3/14 (21%) patients who completed protocol experienced mild withdrawal  3/7 (43%) patient who did not complete protocol discontinued due to withdrawal |
| Raheemullah | Inpatient | Patch | Patient-specific protocols that were all completed within 4 days and included 1 – 3 days of full opioid agonist overlap | 15/15 (100%) experienced mild or minimal withdrawal |
| Schult | Inpatient | Sublingual | 5-day protocol which was completed over a median of 5 days (range 3 – 6 days; IQR 5 – 5 days) in the 54 patients who completed initiation | 2/71 (3%) experienced precipitated withdrawal |
| Sokolski | Inpatient | Patch | 4-day protocol up to 9 mg with at least 3 days of full opioid agonist overlap. After 9mg, additional adjustments of buprenorphine and full opioid agonists occurred as needed.  Initiation was considered complete at first dose of 8 mg SL, which occurred at a mean of 72 (56 – 90) hours. | 1/24 (4%) discontinued due to persistent withdrawal |
| Suen | Outpatient | Sublingual | 4-day protocol with 3 days of full agonist overlap with subsequent adjustments | 1/12 (8%) mild withdrawal  6/12 (50%) no withdrawal |
